# Association of trace elements Aluminum, Copper and Zinc in blood of Alzheimer's disease

**DOI:** 10.1002/alz70860_107018

**Published:** 2025-12-23

**Authors:** Masroor Anwar

**Affiliations:** ^1^ All India Institute of Medical Sciences, New Delhi, Delhi, India

## Abstract

**Background:**

Changes in trace element concentrations in the blood are being wildly being studied due to its role in neurodegenerative effect. The role of metals in the pathogenesis of Alzheimer's disease (AD) is still debated. Although previous research has linked changes in essential metal homeostasis and exposure to environmental heavy metals to the pathogenesis of AD, more research is needed to determine the association between trace elements and AD. Amyloid‐β (Aβ) plaques and neurofibrillary tangles are the main pathological hallmarks of AD. Both Aβ and tau are metal‐binding proteins. Here we performed a quantitative study to analyze the variation in three important elements aluminum (Al), copper (Cu) and zinc (Zn) and compared the metal concentrations between AD patients and healthy controls.

**Method:**

A total of 45 participants were recruited for the study. All the participants underwent comprehensive clinical assessments. Participants were categorized as healthy controls (*N* = 20) and AD (*N* = 25). Elements Al, Cu and Zn levels were analyzed using inductively coupled plasma mass spectrometry, (ICP‐MS, Agilent Technologies, USA). Hindi Mini‐Mental State Examination (HMSE) scores were obtained for the patients and their age and sex‐matched healthy controls.

**Result:**

HMSE score was 12.65 ± 1.334 in patients with AD, and 27.65 ± 0.3529 in control group. Aluminum levels were found to be higher in the AD group (17.05 ± 7.485 µg /L) than in the control group (2.582 ± 0.7399 µg /L) (*p* = 0.0818). Copper levels were higher in AD group 1401 ± 128.4 µg/L than in the control group 1206 ± 67.25 µg/L, (*p* = 0.2251). Interestingly, the concentration of zinc was found to be significantly (*p* = 0.0002) higher in AD (5980 ± 334.2 µg/L) compared to control (8468 ± 466.2 µg/L).

**Conclusion:**

The blood concentration of all the three elements Al, Cu and Zn analyzed in AD patient showed an increased level. Although there was four fold increase in Al but the increase was not found to be significant for both the Al and Cu. Interestingly AD patient had significantly high level of Zn compared to healthy controls.

